# Monocyte and Blood Interleukin-12 Levels Patients with Obstructive Jaundice

**DOI:** 10.1155/1996/96426

**Published:** 1996

**Authors:** W. G. Jiang, M. C. A. Puntis

**Affiliations:** University Department of Surgery University of Wales College of Medicine Heath Park Cardiff CF4 4XN UK

## Abstract

Patients with obstructive jaundice have an increased incidence of peri-operative complications and immune dysfunction. This study was to investigate interleukin (IL)-12 (a cellular immunity stimulant), levels in jaundiced patients. 23 jaundiced patients and 17 controls were studied. There was significantly increased monocyte IL-12 production in jaundice, as measured by an ELISA, compared with that in controls (p<0.01 by Mann-Whitney U test). A similar increase is seen in both benign and malignant jaundice. There was no difference between plasma IL-12 levels in the two groups. It is concluded that in jaundice, monocytes have a significantly increased capacity to release IL-12. This suggests that IL-12 may play a role in the immune dysfunction in jaundiced patients.


*HPB Surgery*, 1996, Vol.9, pp.219–221
Reprints available directly from the publisher
Photocopying permitted by license only

© 1996 OPA (Overseas Publishers Association)
Amsterdam B.V. Published in The Netherlands
by Harwood Academic Publishers GmbH
Printed in Malaysia

### Monocyte and Blood Interleukin-12 Levels in Patients with Obstructive Jaundice

W.G. JIANG and M.C.A. PUNTIS

University Department of Surgery, University of Wales College of Medicine, Heath Park, Cardiff, CF4 4XN, UK

*(Received 26 April 1994)*

Patients with obstructive jaundice have an increased incidence of peri-operative complications and immune dysfunction. This study was to investigate interleukin (IL)-12 (a cellular immunity stimulant), levels in jaundiced patients. 23 jaundiced patients and 17 controls were studied. There was significantly increased monocyte IL-12 production in jaundice, as measured by an ELISA, compared with that in controls ($p<0.01$ by Mann-Whitney U test). A similar increase is seen in both benign and malignant jaundice. There was no difference between plasma IL-12 levels in the two groups. It is concluded that in jaundice, monocytes have a significantly increased capacity to release IL-12. This suggests that IL-12 may play a role in the immune dysfunction in jaundiced patients.

KEY WORDS: Interleukin-12 obstructive jaundice monocytes

### INTRODUCTION

Patients with obstructive jaundice are associated with an increased incidence of perioperative complications, such as renal failure, sepsis, haemorrhagic disorders and endotoxaemia [1–5]. For some years it has been known that these patients have depressed Reticulo-Endothelial system (RES) phagocytic functions [5–8]. It has been reported recently that the secretory functions from monocyte and neutrophils, for example, superoxide production, cytokine secretions, and lysosomal enzyme secretions are increased, and this may be related in some way to a patient's clinical outcome [9–12]. In other studies, it has been shown that patients with jaundice demonstrate lymphocyte activation as indicated by increased IL-2 receptor levels [13].

Interleukin-12, otherwise known as natural killer stimulatory factor (NKSF), is a recently defined cytokine which has major effects on both lymphocytes and NK cells. Both proliferation and function of these two cell types are stimulated by IL-12 [14–19]. The monocyte/macrophage is a key cell type in immune regulation. it produces an array of cytokines including IL-1, IL-6, IL-8, TNF alpha. This cell is also a key contributor to IL-12 in vivo [20]. A markedly raised production of IL-1, IL-6, and TNF from monocytes has been shown to be related to patients' poor prognosis in sepsis, trauma, and burn injuries [21–23].

In order to determine the monocyte cytokine production and the possible relationship to cellular immunity, we have studied the monocyte production and blood level of interleukin-12.

### PATIENTS, METHODS, AND RESULTS

23 patients with biliary obstructions (median age 71 years, 14 male, 9 female) were studied. Of these patients 14 had malignant diseases (cholangiocarcinoma or pancreatic cancer) and the others gall stones or benign biliary strictures. 17 controls were studied (10 had herniae or gall stones without jaundice, 7 colon cancer without liver metastasis, median age 64 years).

Peripheral blood was taken before any clinical intervention. The blood was processed at 4°C. Plasma was obtained by centrifugation at 2500rpm for 25 minutes. Monocytes were separated by a standard Ficoll-Hypaque separation and purified by adherence techniques. The monocyte population was assessed by

*Correspondence to*: WG Jiang and MCA Puntis, University Department of Surgery, University of Wales, College of Medicine, Heath Park, Cardiff CF4 4XN, UK

a non-specific esterase stain (monocyte population was over 90% after adherence). Cells were stimulated *in vitro* with lipopolysaccharide (10μg/ml) for 24 hours and cell free-supernatant were stored at –80°C for later assay.

IL-12 was measured using a specific ELISA with monoclonal 20C2 as capture antibody and R7926 as detecting antibody (Kindly supplied by Genetic Institute, Cambridge, USA). Secondary antibody was horse-reddish peroxidase conjugated IgG (Biorad) and the colour was developed using orthophenylene-diamine (OPD) and absorbance measured at 492 nm on a Tiertek Multiscanner. Recombinant human interleukin-12 was used as standard.

There was a significantly increased IL-12 production in jaundice, 205. 6±2.8pg/ml (mean±SEM), compared with 182.2±4.2pg/ml in controls ($p < 0.01$ by Mann-Whitney U test, figure 1A). When jaundiced patients were separated into groups with either benign or malignant disease, a similar increase is seen in both jaundiced groups.

There was no difference between plasma IL-12 levels in the jaundiced and control groups (170.0±4.5 pg/ml and 171.0±8.0 pg/ml respectively, $p > 0.05$, figure 1B). The differences between benign and malignant in control or that in jaundiced are also not significant. Monocyte IL-12 level in jaundiced patients had no correlation with the plasma bilirubin, bile salt, or aspartate aminotransferase.

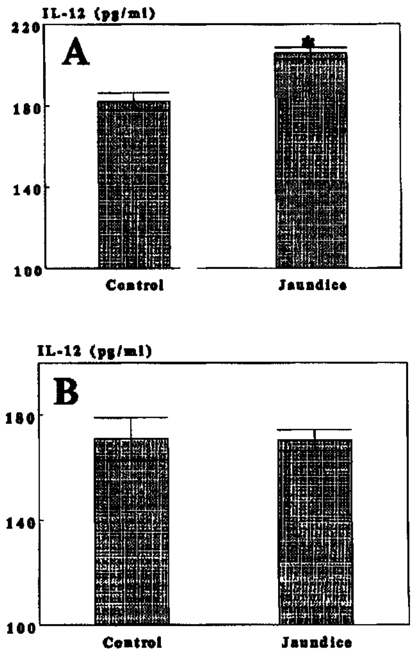

**Figure 1** IL-12 levels in jaundiced patients assayed by ELISA. A: produced by monocytes. B: plasma IL-12. There is a significant increased production from monocytes.

### DISCUSSION

This paper describes a previously unknown phenomenon, patients with biliary obstruction have a raised monocyte interleukin-12 production.

IL-12 is a recently identified cytokine which is produced by lymphocyte and monocyte/macrophage cells, It has major effects on lymphocyte and natural killer (NK) cells (also known as NK cell stimulatory factor, NKSF). This cytokine therefore has an important role in natural and cellular immunity. It has been reported recently that IL-12 may be in the future have some promise as an agent in the treatment of certain infections, such as leishmaniasis [21–22].

Obstructive jaundice is associated with increased incidence of perioperative complications. It is also established that these patients frequently suffer from endotoxaemia [23–26]. Both local and systemic endotoxaemia may therefore be responsible for the immune malfunction in jaundiced patients.

It is not clear by which pathway monocytes increase their production in these patients. It has been reported that IL-12 shares structural homology with IL-6, a proinflammatory cytokines [27,28]. IL-6 production from 'jaundiced' monocyte is reported to be increased [29,30] and this increase may be due to endotoxaemia which occurs in jaundiced patients. Increased IL-12 production may therefore share a similar pathway, this deserves further studies.

In summary, peripheral blood monocyte from obstructive jaundiced patients showed a greatly increased IL-12 production in both benign and malignant aetiologies and this may contribute to the immune malfunction in these patients.

### *Acknowledgement*

We thank Scotia Pharmaceuticals Ltd (UK) for supporting this work. We are grateful for Genetic Institute (Cambridge, USA) for supplying IL-12ELISA kit.

### REFERENCES

1. Pain JA. Cahill CJ. Bailey M.E. (1985). Perioperative complications in obstructive jaundice: therapeutic considerations. *Br J Surg*. **72**: 942–945.
2. Wait RB. Kahng K.V. (1989). Renal failure complicating obstructive jaundice. *Am J Surg*. **157**: 256–263.

3. Allison M.E.M. Prentice CRM. Kennedy A.C. and Blumgart L.H. (1977). Renal function and other factors in obstructive jaundice. *Br J Surg*. **66**: 392–397.
4. Dixon J.M. Armstrong C.P. Duffy R.A. and Davies G.C. (1984). Upper gastrointestinal bleeding. A significant complication after surgery for relief of obstructive jaundice. *Ann Surg*. **199**: 271–275.
5. Halpeax B.N. Biozzi G. Nicol T. and Bilbby D.L.J. (1957). Effect of experimental biliary obstruction on the phagocytic activity of the reticulo-endothelial system. *Nature*. **200**: 503–504.
6. Drivas G. James O. Wardle N. (1976). Study of reticulo-endothelial phagocytic capacity in patients with cholestasis. *Br Med J*, **1**: 1568–1569.
7. Clements W.D. Halliday M.I. McCaigue M.D. Barclay R.G. and Rowlands BJ. (1993). Effects of extrahepatic obstructive jaundice on Kupffer cell clearance capacity. *Arch Surg*. **128**: 200–205.
8. Pain J.A. (1987). Reticuloendothelial function in obstructive jaundice. *Br J Surg*. **74**: 1091–1094.
9. Jiang W.G and Puntis M.C.A. (1993). Monocyte and blood hexosaminidase in patients with obstructive jaundice. *HPB Surg*. **7**: 15–23.
10. Semeraro N. Montemurro P. Chetta G. Altomare DF. Giordano D. and Colucci M. (1989). Increased procoagulant activity of peripheral blood monocyte in human and experimental obstructive jaundice. *Gastroenterol*. **96**: 892– 989.
11. Levy R, Schlaeffer F, Keynan A, Nagauker O, Yaari A, and Sikuler. (1993). Increased neutrophil function induced by bile duct ligation in a rat model. *Hepatology*. **17**: 908 –914.
12. Bemelman M.H.A. Buurman W.A. Gouma D.J. (1993). The presence of TNF and soluble TNF receptors in obstructive jaundice. *Gastroenterol*. **104**: suppl, A665.
13. Wagner F. Assemi C. Lersch C. Hart R. and Classen M. (1990). Soluble interleukin-2 receptor and soluble CD8 in liver cirrhosis and obstructive jaundice. *Clin Exp Immunol*. **82**: 344–349.
14. Kobayashi M. Fitz L. Ryan M. Hewick RM. Clark SC. Chan S. Loudon R. Sherman F. Perussia B. Trinchieri G. (1989): Identification and purification of natural killer cell stimulatory factor (NKSF), a cytokine with multiple biologic effects on human lymphocytes. *J Exp Med* **170**: 827–845.
15. Wolf S.F. Temple P.A. Kobayashi M. Young D. Dicig M. Lowe L. Dzialo R. Fitz L. Ferenz C. Hewick R.M. *et al.* (1991): Cloning of cDNA for natural killer cell stimulatory factor, a heterodimeric cytokine with multiple biologic effects on T and natural killer cells. *J Immunol* **146**: 3074–3081.
16. Gately M.K. Wolitzky A.G. Quinn P.M. Chizzonite R. (1992) Regulation of human cytolytic lymphocyte responses by interleukin-12. *Cell Immunol* **143**: 127–142.
17. Manetti R. Parronchi P. Giudizi M.G. Piccinni M.P. Maggi E. Trinchieri G. Romagnani S. (1993): Natural killer cell stimulatory factor (interleukin 12 [IL-12]) induces T helper type 1 (Th1)-specific immune responses and inhibits the development of IL-4-producing Th cells. *J Exp Med* **177**: 1199 –1204.
18. Perussia B. Chan S.H. D'Andrea A. Tsuji K. Santoli D. Pospisil M. Young D. Wolf S.F. Trinchieri G. (1992): Natural killer (NK) cell stimulatory factor or IL-12 has differential effects on the proliferaion of TCR-alpha beta$^+$, TCR-gamma delta$^+$ T lymphocytes, and NK cells. *J Immunol* **149**: 3495 –3502.
19. Robertson MJ, Soiffer RJ, Wolf SF, Manley TJ, Donahue C. Young D. Herrman S.H. Ritz J. (1992): Response of human natural killer (NK) cells to NK cell stimulatory factor (NKSF): cytolytic activity and proliferation of NK cells are differentially regulated by NKSF. *J Exp Med* **175**: 779–788.
20. D'Andrea A. Rengaraju M. Valiante N.M. Chehimi J. Kubin M. Aste M. Chan S.H. Kobayashi M. Young D. Nickbarg E. *et al.* (1992): Production of natural killer cell stimulatory factor (interleukin 12) by peripheral blood mononuclear cells. *J Exp Med* **176**: 1387–1398.
21. Sypek JP, Chung CL, Mayor SE, Subramanyam JM, Goldman SJ. Sieburth DS, Wolf SF, Schaub RG. (1993): Resolution of cutaneous leishmaniasis: interleukin 12 initiates a protective T helper type 1 immune response. *J Exp Med* **177**: 1797 –1802.
22. Hsieh C.S. Macatonia S.E. Tripp C.S. Wolf S.F. O'Garra A. Murphy K.M. (1993). Development of TH1 CD4+T cells through IL-12 produced by Listeria-induced macrophages. *Science* **260**: 547–549.
23. Greve J.W. Gouma D.J. Soeters P.B. and Burmann W.A. (1990). Suppression of cellular immunity in obstructive jaundice is caused by endotoxin. *Gastroenterol*. **98**: 478– 485.
24. Roughneen PT, Kumar SC, Pellis NR, and Rowlands BJ.(1988). Endotoxaemia and Cholestasis. *Surg Gynecol Obstetr*. **167**: 205–210.
25. Gawley W.F. Gorey T.F. Johnson A.H. Collins P.B. Osborne D.H. Lane B.E. and Collins P.G. (1988). The effect of oral bile salts on serum endotoxin and real functions in obstructive jaundice. *Br J Surg*. **75**: 600.
26. Pain J.A. and Bailey M.E. (1986). Experimental and clinical study of lactulose in obstructive jaundice. *Br J Surg*. **73**: 775 –778.
27. Gearing D.P. Cosman D. (1991). Homology of the p40 subunit of natural killer cell stimulatory factor (NKSF) with the extracellullar domain of the interleukin-6 receptor. *Cell* **66**: 9–10.
28. Merberg D.M. Wolf S.F. Clark S.C. (1992). Sequence similarity between NKSF and the IL-6/G-CSF family. *Immunol Today* **13**: 77–78.
29. Puntis M.C.A. Jiang WG. (1992). Monocytes from obstructive jaundice patients show increased TNF and IL-6 production. *Br J Surg*. **79**: 458–459.
30. Bemelmans M.H.A. Gouma D.J. Greve J.W. Buurma W.A. (1992). Cytokines tumornecrosis factor and interleukin-6 in experimental biliary obstruction in mice. *Hepatology* **15**: 1132–1136.

